# Comparative impact of supine vs prone positioning on dose distribution, acute toxicity, and setup error in postoperative radiotherapy for cervical cancer: a multidimensional propensity-matched cohort study

**DOI:** 10.3389/fonc.2025.1637443

**Published:** 2025-10-22

**Authors:** Nanjie Xiao, Cuiyun Yuan, Tianshu Zhao, Tie Xu, Jiaomei Zhou, Junfang Liao, Miao Peng, Chenbin Liu, Zhijian Chen, Jing Jin

**Affiliations:** Department of Radiation Oncology, National Cancer Center/National Clinical Research Center for Cancer/Cancer Hospital & Shenzhen Hospital, Chinese Academy of Medical Sciences and Peking Union Medical College, Shenzhen, China

**Keywords:** cervical cancer, postoperative radiotherapy, patient positioning, hematologic toxicity, gastrointestinal toxicity

## Abstract

**Background:**

Postoperative radiotherapy is standard for high-risk cervical cancer, but acute toxicities—particularly gastrointestinal and hematologic—remain clinically relevant. Patient positioning may influence organ dose exposure and setup accuracy, yet its multidimensional clinical impact is poorly characterized.

**Methods:**

This retrospective cohort study evaluated patients with cervical cancer treated with postoperative volumetric modulated arc therapy between 2019 and 2022. Propensity score matching (2:1) produced a balanced matched cohort of prone and supine treatments for comparative analyses. Primary endpoints included pelvic organ dose-volume parameters, interfractional setup error, and grade ≥2 hematologic and gastrointestinal toxicities, evaluated using multivariable logistic regression and linear mixed-effects models.

**Results:**

In this single-center retrospective cohort (n = 168), propensity score matching (2:1) yielded 112 balanced patients (prone n = 70; supine n = 42). After matching, target coverage was comparable between positions (PTV_D95: 45.52 Gy vs 45.54 Gy, p = 0.24). The prone group showed higher low-dose exposure in bowel bag and rectum at V5–V15 (e.g., V10 difference −9.84%, 95% CI −17.07 to 1.08; adjusted p = 0.040). Setup error was similar across all axes (p > 0.05). The supine group had significantly higher incidence of leukopenia (92.9% vs 71.4%; p = 0.0073), with prone positioning associated with reduced hematologic toxicity (OR = 14.40, 95% CI 1.60–129.74; p = 0.017). Conversely, diarrhea occurred more often in the prone group (44.3% vs 26.2%, p = 0.070), and supine positioning was protective in multivariable analysis (OR = 0.42, 95% CI 0.17–0.97; p = 0.047).

**Conclusion:**

These findings suggest prone positioning may be preferable for patients with limited hematopoietic reserve, while supine positioning may benefit those with gastrointestinal vulnerability. Positioning choice should be individualized based on toxicity risk and functional anatomy to optimize safety in postoperative cervical cancer radiotherapy.

## Introduction

According to recent data from the International Agency for Research on Cancer (IARC), over 600,000 new cases of cervical cancer and more than 340,000 related deaths were reported globally in 2022, with over 85% of these fatalities occurring in low- and middle-income countries (1). For patients with locally advanced or early-stage disease exhibiting high-risk pathological features, such as positive lymph nodes, positive surgical margins, or deep stromal invasion, adjuvant radiotherapy, including concurrent chemoradiotherapy (CCRT) following radical hysterectomy, is strongly endorsed by major international guidelines, including those from the National Comprehensive Cancer Network (NCCN) and the European Society for Medical Oncology (ESMO), as a standard of care (2, 3). This approach has been shown to significantly reduce pelvic recurrence and improve overall survival. The implementation of advanced radiotherapy techniques, such as intensity-modulated radiotherapy (IMRT) and image-guided radiotherapy (IGRT), has led to substantial progress in optimizing dose conformity and sparing organs at risk (OARs), including the bladder, rectum, bowel, and bone marrow (4, 5). These advancements have enhanced treatment precision and potentially improved patient outcomes. However, treatment-related toxicities, particularly acute gastrointestinal (GI) and hematologic toxicities graded by the Common Terminology Criteria for Adverse Events (CTCAE), remain prevalent and clinically significant (5). Such adverse events can impair treatment adherence, compromise patient-reported quality of life (QoL), and, in severe cases, lead to treatment interruption, which may adversely affect long-term survival outcomes. Consequently, reducing radiation-induced toxicities while maintaining oncological efficacy remains a critical challenge in postoperative radiotherapy for cervical cancer.

Patient positioning, encompassing body posture and immobilization, is central to optimizing postoperative pelvic radiotherapy for cervical cancer, impacting targeting accuracy, organ-at-risk (OAR) sparing, and treatment tolerance. Both prone and supine positions are routinely used (6, 7). Prone positioning with a bellyboard can anteriorly displace small bowel, reducing intermediate-to-high dose exposure and potentially mitigating acute gastrointestinal toxicity (8, 9). Supine positioning, preferred for workflow simplicity, reproducibility, and bladder management, may offer advantages in setup stability and bladder dose control (10, 11). However, most comparative studies are small, single-institution, and methodologically heterogeneous, with few assessing dose–volume metrics, setup accuracy, and multidimensional acute toxicities across key pelvic structures concurrently. We therefore hypothesized that positioning significantly influences pelvic organ dose distribution, setup error, and acute treatment-related toxicities, and that standardized position management may improve safety without compromising target coverage. To test this, we conducted a retrospective analysis using 2:1 propensity score matching to compare prone and supine positioning across multiple endpoints, including OAR dose distribution (bowel bag, rectum, sigmoid colon, and bladder under standardized filling), pelvic bones, setup error, and acute hematologic and gastrointestinal toxicities.

Unlike previous studies that focused on single organs or isolated endpoints, this study employed a comprehensive, multi-organ, and multi-endpoint analytical framework to quantitatively assess the impact of patient positioning during postoperative radiotherapy for cervical cancer. Dosimetric analyses were conducted for key pelvic organs, including the bowel bag, rectum, sigmoid colon, bladder, and pelvic bones, evaluating both dose distribution and irradiated volume. Interfractional setup errors were systematically assessed using periodic image-guided radiotherapy (IGRT) data, and the spectrum of acute treatment-related toxicities was thoroughly characterized. By applying propensity score matching, we effectively balanced the baseline characteristics between the prone and supine groups, thereby minimizing selection bias and enhancing the internal validity and generalizability of the findings. The results of this study are expected to expand the current evidence regarding the clinical implications of positioning strategies for postoperative radiotherapy. Quantitative insights from this study may inform evidence-based positioning protocols, improve workflow precision, enhance patient compliance and quality of life, and serve as a methodological foundation for future large-scale, multicenter prospective cohorts and randomized controlled trials. Ultimately, these findings will contribute to the advancement of more precise and individualized radiotherapy strategies for cervical cancer.

## Materials and methods

### Study design and ethics

This single-center, retrospective cohort study included consecutive patients with cervical cancer who underwent postoperative volumetric modulated arc therapy (VMAT) between November 2019 and October 2022. The inclusion criteria were as follows: (1) histologically confirmed cervical cancer; (2) indication for adjuvant external beam radiotherapy (45.0–50.4 Gy in 25–28 fractions), with or without concurrent chemotherapy, based on National Comprehensive Cancer Network (NCCN) guidelines; and (3) availability of complete radiotherapy planning data and follow-up records. Exclusion criteria were as follows: (1) evidence of distant metastasis; (2) prior pelvic radiotherapy; and (3) active colorectal disease, including but not limited to inflammatory bowel disease and other obstructive or bleeding conditions. The study protocol was approved by the institutional ethics committee (approval no. JS2024-32-1) and the requirement for informed consent was waived. All patient data were anonymized prior to analysis.

### CT simulation and positioning

The CT simulation was conducted utilizing a Discovery 590 RT scanner (GE Healthcare, Waukesha, WI, USA) with a slice thickness of 5 mm. Patients positioned prone were immobilized using a belly board system, whereas those positioned supine were immobilized with a thermoplastic mask and a flat tabletop. Bowel preparation involved rectal evacuation and standardized bladder filling. Each patient was instructed to consume 500 mL of water and wait 30 minutes prior to the simulation and each treatment session. Ultrasonography was employed to confirm that the bladder volume ranged between 120 and 150 mL before treatment commencement. The scan range extended from the T10 vertebral body to 5 cm below the ischial tuberosity. The quality of the acquired images was verified to be sufficient for precise delineation of the pelvic structures. Prone patients were immobilized using a belly-board system with pelvic fixation; supine patients were immobilized using a thermoplastic mask and flat tabletop. Bladder filling and bowel preparation were standardized. Daily on-treatment verification (CBCT) was performed during the first five fractions and at least weekly thereafter to ensure reproducibility.

### Target volume and organ-at-risk delineation

The target volumes were delineated in accordance with a harmonized protocol integrating elements from the RTOG 0418, 0529, and 0822 guidelines. The clinical target volume (CTV) encompassed the postoperative tumor bed, vaginal cuff, and regional lymphatic drainage areas, including the obturator, internal and external iliac, and presacral regions. In patients exhibiting high-risk pathological features, the upper boundary of the CTV was extended superiorly to the level of the renal vessels, a configuration defined as extended-field irradiation in this study. A uniform 0.5 cm expansion was applied to generate the planning target volume (PTV). Organs at risk (OARs) located within 2 cm of the PTV, including the bladder, rectum, sigmoid colon, and femoral heads, were contoured in accordance with RTOG guidelines. The sigmoid colon was delineated as a distinct structure based on anatomical boundaries. The bowel bag was contoured following the method described by Robyn et al (12).

### Radiotherapy planning

All radiotherapy plans were developed using the Eclipse treatment planning system (version 7.3.10; Varian Medical Systems) employing dual-arc volumetric modulated arc therapy (VMAT). The prescribed dose ranged from 45.0 to 48.6 Gy in 25–27 fractions, determined at the discretion of the treating physician based on individual risk factors and clinical judgment. The planning objectives stipulated that at least 95% of the planning target volume (PTV) received 100% of the prescribed dose, with a maximum dose not exceeding 107%. Dose constraints for organs at risk (OARs) were based on the Quantitative Analyses of Normal Tissue Effects in the Clinic (QUANTEC) guidelines. Dose-volume histogram (DVH) parameters were extracted for each OAR at 5 Gy intervals from V5 to V55 and reported as both absolute volume (in cm^3^) and relative volume (percentage of total OAR volume).

### Image guidance and setup error assessment

Cone-beam computed tomography (CBCT) was performed daily for the first five treatment fractions and weekly thereafter. Three-dimensional deviations in the lateral (X), longitudinal (Y), and vertical (Z) axes were recorded for each session to evaluate interfractional setup error. Systematic and random error decomposition was not performed. CBCT datasets with missing or unusable data were excluded from the final analysis.

### Clinical data collection and toxicity assessment

The body mass index (BMI) was determined by dividing weight in kilograms by height in meters squared (kg/m^2^), with weight measurements taken each Wednesday morning under fasting conditions. Complete blood counts were conducted weekly before and during radiotherapy using a standardized laboratory platform. The lowest recorded values during treatment were utilized to evaluate hematologic toxicity, including leukocyte, hemoglobin, platelet, and neutrophil counts. Toxicities were graded according to the Common Terminology Criteria for Adverse Events (CTCAE), version 5.0. Hematological toxicity was defined as grade ≥2 if any of the following thresholds were met: leukopenia (WBC < 3 × 10^9^/L), anemia (Hb < 100 g/L), thrombocytopenia (PLT < 75 × 10^9^/L), or neutropenia (NEU < 1.5 × 10^9^/L). Gastrointestinal toxicity was assessed by the maximum daily bowel movement frequency, with ≥3 stools/day classified as grade ≥2 diarrhea. Other gastrointestinal symptoms were not included in the toxicity analysis.

### Propensity score matching

To mitigate baseline confounding between the treatment groups, propensity score matching (PSM) was executed using a multivariable logistic regression model. Covariate selection was informed by prior literature and expert clinical consensus and included age, pathological stage, histological subtype, receipt of neoadjuvant chemotherapy, receipt of concurrent chemoradiotherapy, radiation field size, use of brachytherapy, body mass index (BMI), PTV_D95, PTV_mean, and baseline hematologic indices (white blood cell count, hemoglobin, platelet count, and neutrophil count). A 2:1 nearest-neighbor matching algorithm without replacement was implemented using the MatchIt package in R (version 4.3.1), with a caliper width of 0.45 selected to balance sample retention and matching quality based on a previously published methodology. Matching balance was evaluated using standardized mean differences (SMD), with values <0.1 considered indicative of acceptable balance.

### Statistical analysis

#### Dosimetric comparisons

For each dose-volume threshold from V5 to V55, the median and interquartile range (IQR) of the relative irradiated volume (defined as the percentage of the organ volume receiving at least X Gy) were calculated for both the matched prone and supine groups. Group comparisons were performed using the Wilcoxon rank-sum test. P-values were adjusted for multiple comparisons using the Benjamini–Hochberg false discovery rate (FDR) method. The magnitude of the group differences was quantified using Cliff’s delta (Cliff’s Δ), with negative values indicating lower values in the prone group. The between-group median differences and their 95% bootstrap confidence intervals were estimated using 2,000 resamples.

### Setup error analysis

Interfractional setup errors (X, Y, and Z axes) were analyzed using linear mixed-effects models (lme4 package), with treatment position, fraction number, and their interaction as fixed effects, and patient ID as a random intercept. The model assumptions, including residual normality and variance structures, were verified.

### Acute toxicity analysis

To identify potential factors associated with acute toxicity, separate logistic regression models were constructed using grade ≥2 hematologic toxicity and grade ≥2 diarrhea as dependent variables. Univariable logistic regression was first performed for all clinical characteristics and organ-at-risk dose–volume histogram (DVH) parameters. Variables with a p-value of less than 0.20 in the univariable analysis were subsequently incorporated into multivariable logistic regression models. A stepwise backward elimination method was employed for variable selection, and the final models were selected based on the lowest Akaike Information Criterion (AIC) value to optimize model fit and parsimony. Variable selection was entirely based on statistical criteria, with no covariates being forced into the model. Hematologic toxicity and diarrhea were modeled independently. Toxicities were clinician-graded per CTCAE v5.0; validated patient-reported outcomes were not available due to the retrospective nature of the study.

#### Other statistical tests

Normally distributed continuous variables were analyzed using the Student’s t-test, while non-normally distributed variables were assessed using the Wilcoxon rank-sum test. Categorical variables were compared using the chi-squared test or Fisher’s exact test, as appropriate. All analyses were performed using R software (version 4.3.1) with the following packages: MatchIt, tableone, rstatix, lme4, and survival. All tests were two-sided, with p-values less than 0.05 considered statistically significant. All statistical analyses were conducted using a complete case dataset, with observations containing missing values excluded from the analysis.

## Results

**Figure 1:**
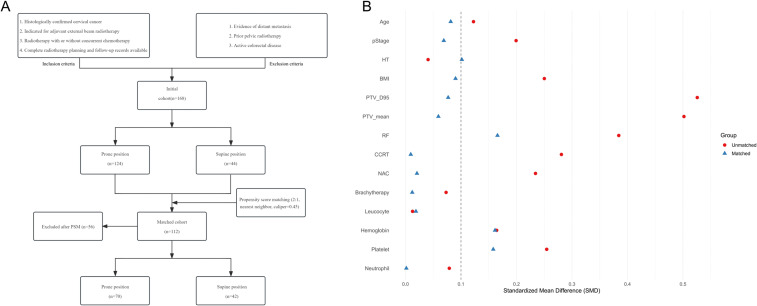
Patient selection flow and baseline covariate balance before and after propensity score matching. **(A)** Flow chart of patient inclusion and exclusion. The diagram summarizes the process of patient screening, exclusion criteria, and the final number of cases included in the analysis. **(B)** Love plot showing the standardized mean differences (SMDs) for baseline covariates before and after matching. Each point represents the SMD for an individual covariate; values less than 0.1 after matching are considered indicative of adequate balance between groups. PSM = propensity score matching; SMD = standardized mean difference.

**Figure 2:**
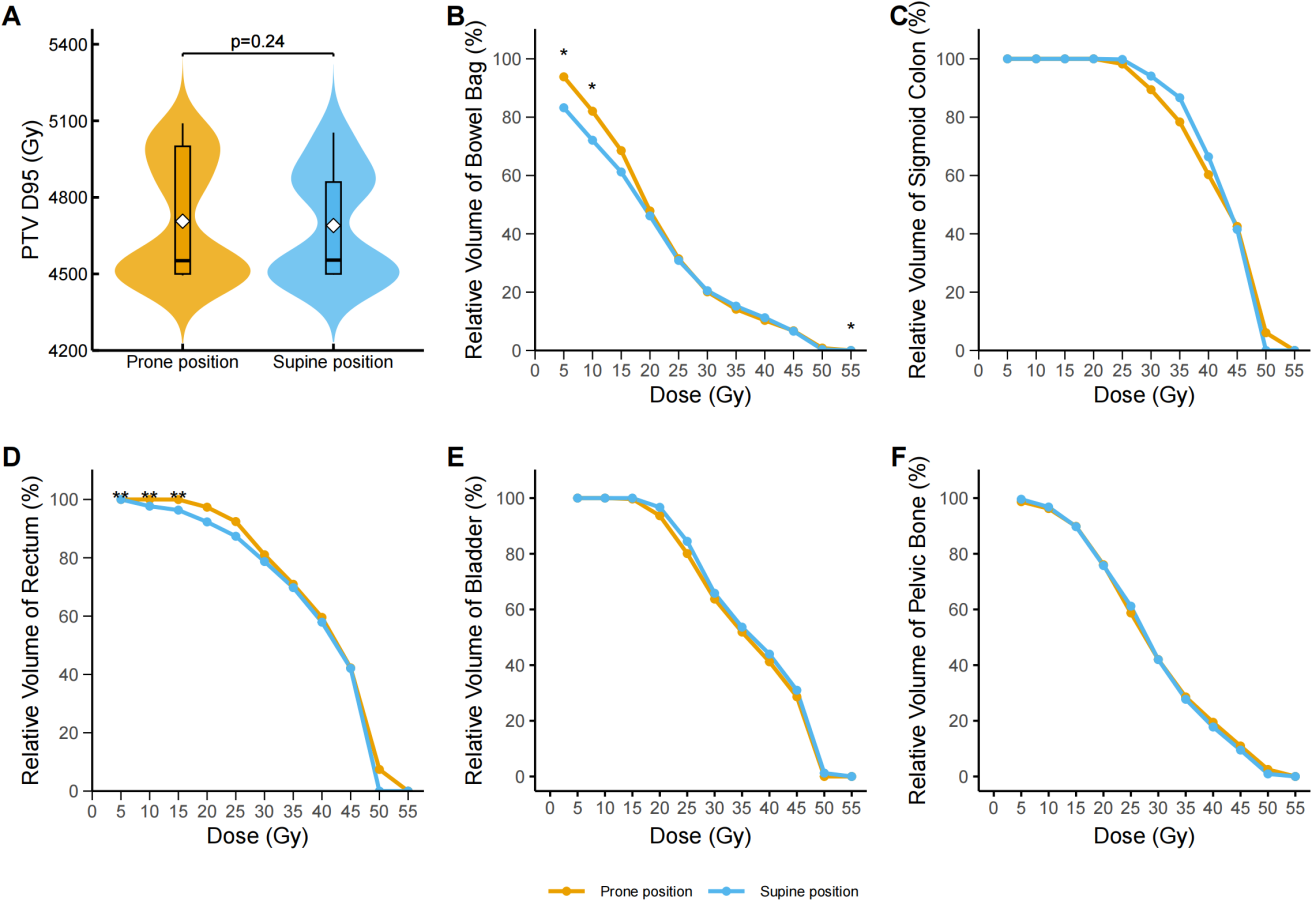
Dose–volume analysis of pelvic organs and target volume in prone and supine positions after propensity score matching. **(A)** Distribution of the dose covering 95% of the planning target volume (PTV D95, Gy) in the prone and supine groups. Violin plots display the full distribution with embedded boxplots showing median and interquartile range; white diamonds represent the mean. No significant difference was observed (p = 0.24, Wilcoxon signed-rank test). **(B)** Dose–volume histogram (DVH) curves for the bowel bag. The supine group exhibited significantly lower irradiated volumes at V5 (p = 0.041), V10 (p = 0.041), and V55 (p = 0.041) after false discovery rate (FDR) correction. Data represent group medians; asterisks denote statistical significance. **(C)** DVH curves for the sigmoid colon. No statistically significant differences were observed at any dose level (V5–V55) following FDR correction. **(D)** DVH curves for the rectum. The supine group had significantly lower relative volumes at V5 (adjusted p = 0.0013), V10 (adjusted p = 0.0011), and V15 (adjusted p = 0.0032) after FDR correction; asterisks denote statistical significance. **(E)** DVH curves for the bladder. No statistically significant differences were found across any dose levels (V5–V55) following FDR correction. **(F)** DVH curves for the pelvic bone. No statistically significant between-group differences were observed at any dose point after FDR correction. *p < 0.05, **p < 0.01, ***p < 0.001. The asterisk indicates statistical significance in the relative volume at a specific dose level for a given organ-at-risk.

A total of 168 patients were included in the study, comprising 124 patients in the prone position group and 44 patients in the supine position group. Patients in the prone group exhibited significantly higher PTV_D95 and PTV_mean values compared to those in the supine group (48.60 vs. 45.26 Gy, p = 0.001; 50.60 vs. 47.66 Gy, p = 0.007). Additionally, a greater proportion of patients in the supine position received extended-field irradiation (25.0% vs. 10.5%; p = 0.02) (**Table 1**). After propensity score matching for clinical and dosimetric variables, including PTV_D95 and PTV_mean, 112 patients were retained, with 70 in the prone group and 42 in the supine group (**Figure 1A**). The post-matching baseline characteristics were generally well balanced between the two groups. Standardized mean differences (SMDs) were less than 0.1 for most variables, except for hemoglobin (SMD = 0.16), platelet count (SMD = 0.16), and irradiation range (SMD = 0.17); no covariate exceeded an SMD of 0.2 (**Table 1**, **Figure 1B**). Following matching, there was no statistically significant difference in PTV_D95 between the prone and supine groups (median [IQR]: 45.52 [44.94–50.90] Gy vs. 45.54 [45.00–50.54] Gy; p = 0.24) (**Table 1**, **Figure 2A**, **Supplementary Figure S1**). No other baseline variables differed significantly between the groups (all p > 0.2).

**Table 1:** Patients’ baseline characteristics before and after propensity-score matching.

Characteristics	Original cohort (n =168 )		P value	SMD	Matched cohort (n =112 )		P value	SMD
	Prone position (n=124)	Supine position (n=44)			Prone position (n=70)	Supine position (n=42)		
Age,year (mean ± SD)	53.01 ± 10.08	51.93 ± 8.77	0.53	0.12	52.66 ± 10.09	51.88 ± 8.91	0.68	0.08
Pathologic stage			0.52	0.20			0.94	0.07
I	46(37.1%)	15(34.1%)			27 (38.6%)	15 (35.7%)		
II	36(29.0%)	10(22.7%)			15 (21.4%)	10 (23.8%)		
III	42(33.9%)	19(43.2%)			28 (40.0%)	17 (40.5%)		
Histological type			0.97	0.04			0.87	0.10
Squamous carcinoma	99(79.8%)	35(79.5%)			57 (81.4%)	34 (81.0%)		
Adenocarcinoma	15(12.1%)	5(11.4%)			8 (11.4%)	4 (9.5%)		
Other	10(8.1%)	4(9.1%)			5 (7.1%)	4 (9.5%)		
BMI,kg/m2 (median,range)	23.31 (17.83-33.20)	21.83 (17.33-31.24)	0.03	0.25	22.9 (17.8–29.6)	22 (17.3–31.2)	0.21	0.09
Leucocyte (median,range)	4.68 (1.48-14.63)	4.68 (1.52-23.85)	0.42	0.01	4.56 (1.48–10.4)	4.67 (1.52–23.9)	0.47	0.02
Hemoglobin (median,range)	108.00 (82-132.00)	106.50 (92-128.00)	0.27	0.16	108 (87–132)	106 (92–128)	0.33	0.16
Platelet (median,range)	241.50 (80-551)	223.50 (72-429)	0.20	0.25	233 (80–403)	232 (72–429)	0.38	0.16
Neutrophil (median,range)	2.67 (0.1-13.55)	2.23 (0.26-17.39)	0.25	0.08	2.63 (0.1–7.95)	2.23 (0.26–17.4)	0.43	0.002
Neoadjuvant chemotherapy			0.21	0.23			1.00	0.02
Yes	74(59.7%)	31(70.5%)			49 (70.0%)	29 (69.0%)		
No	50(40.3%)	13(29.5%)			21 (30.0%)	13 (31.0%)		
Concurrent chemotherapy			0.10	0.28			1.00	0.01
Yes	50(40.3%)	24(54.5%)			37 (52.9%)	22 (52.4%)		
No	74(59.7%)	20(45.5%)			33 (47.1%)	20 (47.6%)		
Irradiation range			0.02	0.38			0.54	0.17
Standard field	111(89.5%)	33(75.0%)			58 (82.9%)	32 (76.2%)		
Extension field	13(10.5%)	11(25.0%)			12 (17.1%)	10 (23.8%)		
Brachytherapy			0.66	0.07			1.00	0.01
Yes	19(15.3%)	8(18.2%)			13 (18.6%)	8 (19.0%)		
No	105(84.7%)	36(81.8%)			57 (81.4%)	34 (81.0%)		
Dmean_PTV(Gy) (median,range)	50.60 (46.36-53.43)	47.66 (46.35-52.33)	0.007	0.50	47.40 (46.36–53.23)	47.85 (46.35–52.33)	0.87	0.06
D95_PTV(Gy) (median,range)	48.60 (44.89-51.43)	45.26 (45.00-50.54)	0.001	0.53	45.52 (44.94–50.90)	45.54 (45.00–50.54)	0.24	0.08

SMD, standardized mean difference.

### Dosimetric comparison

Across all dose-volume thresholds (V5–V55), no significant differences were observed between the prone and supine groups in terms of the absolute irradiated volume of the bowel bag (**Table 2**). However, dose–volume histogram (DVH) analysis revealed significant differences in the relative irradiated volumes at V5, V10, and V55. Specifically, the V5 volume was significantly higher in the prone group than in the supine group (median difference −10.74%, 95% CI −17.05% to −0.96%; Cliff’s Δ = 0.32; FDR-adjusted p = 0.040). A similar pattern was observed at V10 (median difference: −9.84%, 95% CI: −17.07% to 1.08%; Cliff’s Δ = 0.30; adjusted p = 0.040). While a statistically significant difference was observed at V55 (median difference 0.00%, 95% CI 0.00 to 0.00; Cliff’s Δ = −0.15; adjusted p = 0.040), the magnitude of this difference was negligible (**Figure 2B**). Dose-volume histogram (DVH) analysis of the sigmoid colon revealed no statistically significant differences in the relative irradiated volume between the two groups at any dose level from V5 to V55 (all FDR-adjusted p ≥ 0.05) (**Figure 2C**). Similarly, no significant differences were detected in the absolute irradiated volume of the sigmoid colon across all dose levels (all p > 0.05) (**Table 2**). In the rectum, the supine group demonstrated significantly lower irradiated volumes at V10 and V15 compared to the prone group (**Figure 2D**). At V10, the median relative volume was 97.69% in the supine group versus 100.00% in the prone group (median difference: −2.31%, 95% CI: −7.49% to −2.39%; Cliff’s Δ = 0.41; adjusted p = 0.001). At V15, the difference further increased (96.34% vs. 99.97%; median difference, −3.63%; 95% CI, −8.79% to −3.26%; Cliff’s Δ = 0.37; adjusted p = 0.003). Although both groups had a median V5 value of 100.00%, the distributions differed significantly (Cliff’s Δ = 0.31; unadjusted p = 0.001). No significant differences were found in the absolute irradiated volume of the rectum between the two groups at any dose level (all p > 0.05) (**Table 2**). Regarding the bladder, the prone group consistently exhibited lower relative irradiated volumes across V5–V55; however, none of these differences reached statistical significance after FDR correction (adjusted p ≥ 0.05) (**Table 2**, **Figure 2E**). Prior to adjustment, the supine group showed slightly higher relative pelvic volumes at V5 and V10 (V5: 99.50% [IQR 98.43%–99.85%] vs. 98.95% [94.66%–99.65%], p = 0.01; V10: 96.57% [95.39%–98.37%] vs. 96.28% [89.90%–97.50%], p = 0.01), but these differences were not significant after correction (**Figure 2F**).

**Table 2:** Dose–volume parameters in the prone and supine groups after propensity score matching.

Dose-volume parameters (median [IQR])	Bowel bag		P	Sigmoid		P	Rectum		P	Bladder		P	Pelvic bone		P
	Prone station (n=70) (cc)	Supine station (n=42) (cc)		Prone station (n=70) (cc)	Supine station (n=42) (cc)		Prone station (n=70) (cc)	Supine station (n=42) (cc)		Prone station (n=70) (%)	Supine station (n=42) (%)		Prone station (n=70) (%)	Supine station (n=42) (%)	
V5	1968.56 (1559.49–2208.31)	1897.76 (1542.94–2277.11)	0.80	57.17 (41.94–94.71)	65.92 (38.51–99.55)	0.64	37.01 (28.85–52.71)	37.81 (31.07–44.25)	0.61	100 (100–100)	100 (100–100)	0.41	98.95 (94.66–99.65)	99.5 (98.43–99.85)	0.01
V10	1821.44 (1427.64–2008.04)	1708.33 (1412.39–2041.62)	0.92	57.17 (41.94–94.71)	65.92 (38.51–99.55)	0.64	36.66 (28.85–51.5)	36.92 (29.04–44.16)	0.50	100 (100–100)	100 (100–100)	0.14	96.28 (89.9–97.5)	96.57 (95.39–98.37)	0.01
V15	1497.57 (1233.19–1706.08)	1451.88 (1302.16–1761.1)	0.88	57.17 (41.94–94.71)	64.85 (38.51–98.98)	0.68	35.88 (28.03–51.03)	36.25 (28.35–42.94)	0.49	99.61 (95.98–100)	100 (98.85–100)	0.10	89.9 (83.2–93.84)	89.74 (87.58–93.49)	0.31
V20	1076.36 (931.68–1265.71)	1096.18 (960.1–1304.69)	0.69	57.17 (40.64–93.67)	62.27 (38.39–90.19)	0.74	33.17 (27.67–49.67)	35.03 (27.16–42.5)	0.57	93.36 (86.76–98.58)	96.69 (88.43–98.9)	0.21	76.21 (70.83–83.48)	75.77 (71.7–81.38)	0.97
V25	745.92 (645.24–904.71)	769.11 (660.7–898.98)	0.58	56.59 (38.29–85.6)	59.39 (34.61–89.4)	0.89	32.09 (25.61–45.96)	33.06 (26.18–40.28)	0.75	78.1 (70.65–89.8)	84.46 (74.83–91.42)	0.25	59.27 (55.78–65.97)	61.19 (56.52–64.92)	0.56
V30	533.62 (424.29–638.74)	547.54 (467.15–653.46)	0.60	47.51 (32.69–69.64)	51.57 (32.49–83.25)	0.75	30.1 (23.72–40)	31.06 (23.71–36.57)	0.95	63.69 (56.29–70.52)	66.2 (57.68–71.59)	0.33	42.06 (39.2–46.54)	41.97 (38.95–47.38)	0.99
V35	398 (313.51–477.31)	400.11 (339.07–477.79)	0.64	41.23 (27.81–61.65)	45.12 (29.96–75.23)	0.70	26.44 (21.24–36.45)	27.59 (21.92–33.87)	0.85	51.79 (45.58–56.35)	53.88 (46.32–60.29)	0.28	28.57 (25.63–32.42)	27.72 (25.05–30.8)	0.39
V40	305.85 (235.07–363.51)	309.79 (242.73–350.74)	0.93	33.92 (23.58–47.74)	34.81 (21.22–59.26)	0.76	20.96 (17.57–30.79)	23.84 (18.14–30.9)	0.63	41.56 (35.72–46.02)	44.79 (37.27–48.84)	0.20	19.47 (16.47–22.2)	17.78 (16.29–19.95)	0.14
V45	199.55 (154.3–253.4)	188.81 (138.39–254.23)	0.61	21.22 (13.76–32.01)	22.19 (13.89–37.55)	0.65	15.33 (11.87–23.48)	17.01 (13.16–23.16)	0.53	28.78 (23.8–34.43)	30.98 (27.24–37.47)	0.15	10.95 (8.89–14.55)	9.37 (7.88–11.36)	0.06
V50	0 (0–93.5)	7.2 (0–100.61)	0.35	0 (0–5.86)	1.38 (0–3.73)	0.39	0 (0–6.89)	0 (0–7.82)	0.58	0 (0–15.93)	1.48 (0–17.54)	0.22	2.92 (0–7.05)	0.82 (0–3.29)	0.13
V55	0 (0–0)	0 (0–0)	0.86	0 (0–0)	0 (0–0)	0.82	0 (0–0)	0 (0–0)	0.42	0 (0–0)	0 (0–0)	0.17	0 (0–0)	0 (0–0)	0.31

### Setup error

**Figure 3:**
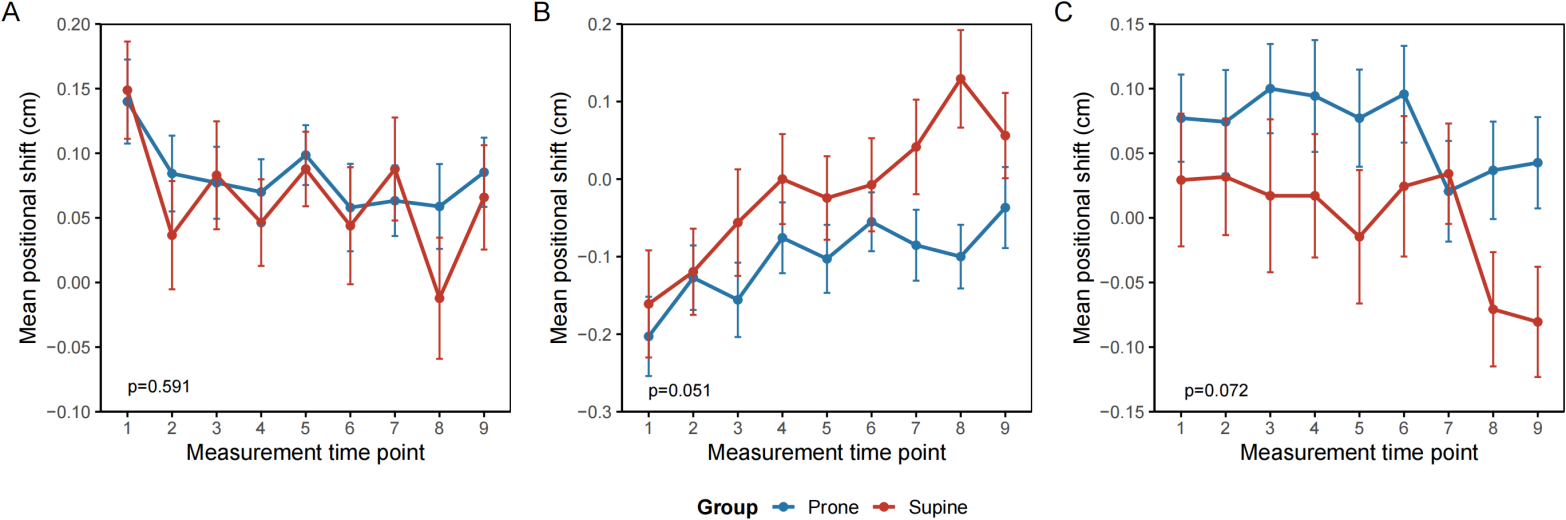
Repeated measures of interfractional setup error by treatment position. **(A)** Vertical axis (X) **(B)** Longitudinal axis (Y) **(C)** Lateral axis (Z). Mean interfractional setup deviations (cm) are plotted across treatment fractions (1–9) for patients in the prone (blue) and supine (red) positions. Error bars represent the standard error of the mean. No significant differences were observed between groups across all spatial directions. Trends over time reflect day-to-day variation in patient alignment and organ motion. All measurements were derived from cone-beam computed tomography (CBCT) image guidance.

Analysis of setup error using a generalized linear mixed-effects model (GLMM) revealed no statistically significant difference in setup error along the x-axis (vertical direction) between the prone and supine groups. The mean displacement was 0.081 cm (95% CI: 0.046–0.117) in the prone group and 0.065 cm (95% CI: 0.019–0.112) in the supine group (p = 0.591). Time, defined as the sequential number of image-guided radiotherapy sessions, was a significant factor affecting displacement along the x-axis (p = 0.006). The interaction between group and time was not significant (p = 0.85) (**Figure 3A**). Along the y-axis (longitudinal direction), the mean displacement was −0.102 cm (95% CI, −0.154 to −0.049) in the prone group and −0.016 cm (95% CI, −0.084 to 0.053) in the supine group. The difference between groups was not statistically significant (p = 0.051). Time remained a significant factor (p < 0.001), whereas the group–time interaction was not significant (p = 0.55) (**Figure 3B**). On the z-axis (lateral direction), the mean displacement was 0.071 cm (95% CI, 0.023–0.117) in the prone group and −0.001 cm (95% CI, −0.063–0.060) in the supine group. No significant differences were observed between groups (p = 0.072). Neither time (p = 0.153) nor the interaction term (p = 0.74) was statistically significant (**Figure 3C**).

### Hematologic toxicity

**Figure 4:**
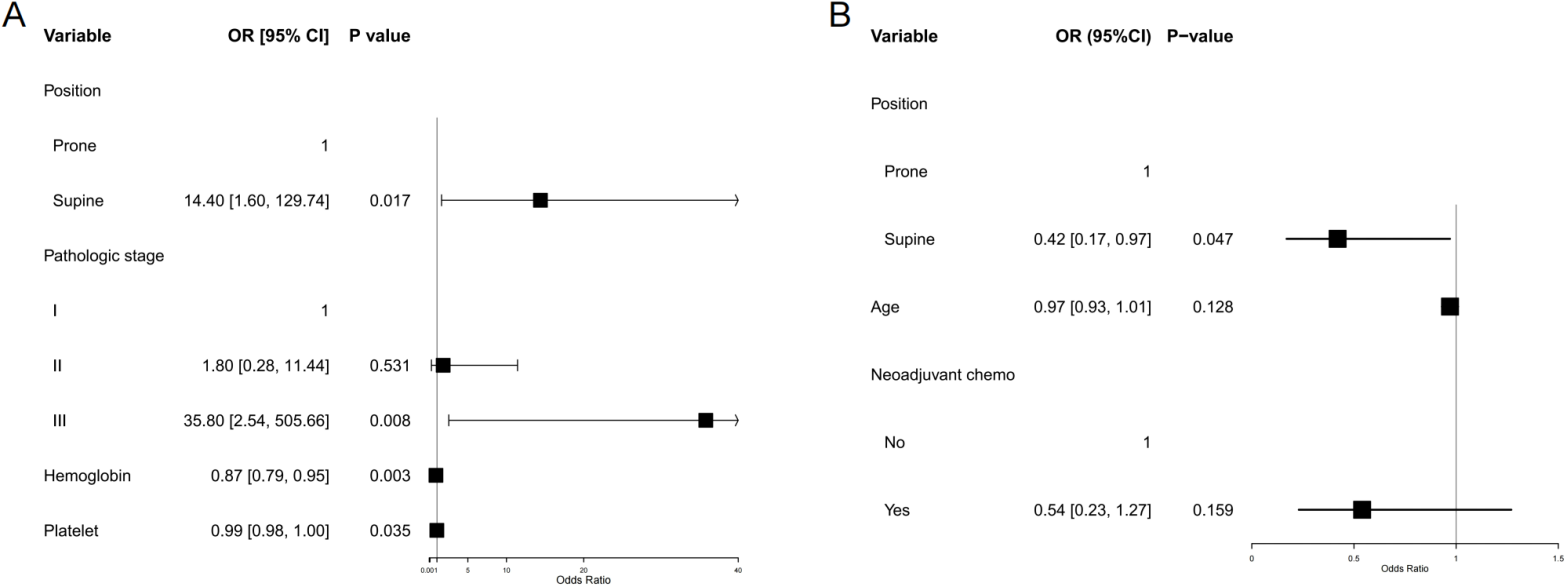
Forest plot of multivariable-adjusted associations between treatment position and acute toxicities. **(A)** Hematologic toxicity (grade ≥2). **(B)** Diarrhea (grade ≥2). Forest plots show adjusted odds ratios (ORs) with 95% confidence intervals (CIs) for the association between radiotherapy position (prone vs supine) and the risk of major acute toxicities. Multivariable logistic regression was performed for each endpoint, adjusting for relevant baseline clinical characteristics and dose–volume parameters. Prone positioning was independently associated with a lower risk of hematologic toxicity, while supine positioning was associated with a reduced risk of acute diarrhea. Vertical dashed lines indicate the null value (OR = 1.0); error bars represent 95% Cis.

In the matched population of postoperative cervical cancer patients, the incidence of grade ≥2 leukopenia was significantly higher in the supine group compared to the prone group (92.9% vs. 71.4%; absolute difference, 21.4%; 95% CI 6.4–36.5; p = 0.0073). No statistically significant differences were observed between the two groups regarding the incidence of grade ≥2 neutropenia, anemia, or thrombocytopenia (all p > 0.05). When evaluating the composite incidence of any hematologic toxicity (grade ≥2 in any of the four parameters), the supine group again exhibited a significantly higher rate (95.2% vs. 81.4%; absolute difference 13.8%, 95% CI 0.7–26.9; p = 0.046) (**Table 3**). Multivariable logistic regression analysis indicated that patients receiving radiotherapy in the supine position had a significantly elevated risk of developing grade ≥2 hematologic toxicity compared to those treated in the prone position (odds ratio [OR] = 14.40, 95% confidence interval [CI] 1.598–129.738; p = 0.017) (**Figure 4A**). For each 1 g/L increase in baseline hemoglobin level, the risk of hematologic toxicity decreased by 13% (OR = 0.87, 95% CI 0.794–0.953; p = 0.0027). Conversely, for each 1 × 10^9^/L decrease in platelet count, the risk increased by 1.3% (OR = 0.987, 95% CI 0.976–0.999; p = 0.0347). Compared to patients with pathological stage I disease, those with stage III disease had a significantly higher risk of toxicity (OR = 35.81, 95% CI 2.535–505.663; p = 0.008), whereas no significant difference was observed for patients with stage II disease.

**Table 3:** Comparison of grade ≥2 acute toxicities between matched postoperative cervical cancer patients.

Toxicity	Supine station (n=42) (%)	Prone station (n=70)(%)	Absolute difference (Δ, %)	95% CI	P-value
Leucocyte (≥2)	39 (92.9%)	50 (71.4%)	21.4	6.4 - 36.5	0.007
Hemoglobin (≥2)	19 (45.2%)	31 (44.3%)	1	–19.0 - 20.9	1.00
Platelet (≥2)	5 (11.9%)	4 (5.7%)	6.2	–6.9 - 19.3	0.292
Neutrophil (≥2)	28 (66.7%)	39 (55.7%)	11	–9.4 - 31.3	0.320
Hematologic toxicity (≥2)	40 (95.2%)	57 (81.4%)	13.8	0.7 - 26.9	0.046
Diarrhea (≥2)	11 (26.2%)	31 (44.3%)	–18.1	–37.7 - 1.5	0.070

Δ = absolute difference between groups. 95% CI calculated via bootstrapping. p-values are based on Pearson’s χ^2^ test unless otherwise noted. Fisher’s exact test was used when expected cell counts were <5. Hematologic toxicity indicates the presence of grade ≥2 toxicity in any of the following: leucocyte, hemoglobin, platelet, or neutrophil. Toxicities graded per CTCAE v5.0.

### Gastrointestinal toxicity

Among the matched postoperative cervical cancer patients, the incidence of diarrhea with more than three episodes per day was 26.2% in the supine group and 44.3% in the prone group, resulting in an absolute difference of −18.1% (95% CI, −37.7 − 1.5; p = 0.07). Although the prone position was associated with a higher tendency for diarrhea, the difference was not statistically significant (**Table 3**). In the multivariable logistic regression analysis, patients in the supine position had a significantly lower risk of diarrhea than those in the prone position (OR = 0.42, 95% CI 0.17–0.97; p = 0.047). Neither age nor the receipt of neoadjuvant chemotherapy was significantly associated with the risk of diarrhea (**Figure 4B**).

## Discussion

This study systematically evaluated the differences between the prone and supine positions during postoperative radiotherapy for cervical cancer in terms of target dose distribution, normal tissue exposure, hematologic and gastrointestinal toxicity, and setup errors. Propensity score matching and multivariate statistical adjustments were employed to minimize confounding factors. The results demonstrated that despite overall comparable dose distributions, the choice of treatment position significantly affected low-dose organ exposure and the incidence of specific toxicities. These findings suggest that patient positioning is not a neutral technical variable and should be considered an integral component of individualized radiotherapy planning.

Following propensity score matching, no statistically significant differences were identified between the supine and prone groups concerning key dosimetric parameters, including the dose received by 95% of the planning target volume (PTV_D95) and the mean PTV dose (PTV_mean). This indicates comparable target coverage and overall dose homogeneity between the two positioning strategies. Further analysis of dose–volume histograms (DVHs) revealed that, although the absolute irradiated volumes of the bowel bag and rectum did not differ significantly between groups, the relative volume receiving low-dose exposure (V5–V15) was significantly greater in the prone group. This phenomenon may be attributed to the gravitational shift of mobile abdominal organs, particularly the small bowel, toward the anterior abdominal wall in the prone position (13). This anterior displacement increases the likelihood that these structures fall within the peripheral regions of the radiation field, where beam penumbra and scatter contribute to the so-called “low-dose bath”. Conversely, during supine positioning, the bowel may shift posteriorly toward the spine or sacrum, potentially reducing exposure to these low-dose regions. Such a pattern of low-dose redistribution has been previously reported in studies of rectal cancer and gynecologic malignancies, with changes predominantly observed in the V5–V15 range (14, 15). Variations in low-dose exposure may not directly cause high-grade toxicity; however, the cumulative low-dose bath and scatter could plausibly contribute to subclinical symptoms through chronic mucosal inflammation and neuroimmune modulation (16). Given that this linkage remains largely associative, prospective studies incorporating quantitative correlative endpoints are needed to establish causality. These findings underscore the potential clinical relevance of monitoring V5–V15 parameters during treatment planning and toxicity risk assessment.

With respect to setup errors, both the prone and supine positions demonstrated comparable accuracy across all three translational directions, with deviations remaining within clinically acceptable thresholds. These findings are consistent with results from large-cohort studies in pelvic radiotherapy that have demonstrated comparable setup accuracy across different treatment positions (17, 18). In the present study, the largest deviations were observed in the longitudinal (superior–inferior) direction, however, these did not reach clinical significance. Treatment duration emerged as an independent factor influencing setup accuracy, potentially reflecting dynamic changes, such as patient fatigue, positional instability, and variations in organ filling. Notably, similar trends of increasing setup errors over the course of treatment have also been observed in postoperative rectal cancer patients (19). While some reports have suggested that the prone position may be associated with larger systematic errors, primarily due to inter-individual anatomical variability and respiratory motion, this study incorporated standardized immobilization protocols and routine image-guided verification, effectively mitigating such concerns. These results support the feasibility and safety of prone positioning in the context of modern radiotherapy techniques (20–22). Daily verification imaging supported the reproducibility and operational feasibility of prone positioning in routine practice.

Regarding treatment-related toxicity, the prone position demonstrated a significant advantage in terms of hematologic preservation. Patients treated in the supine position exhibited a markedly higher incidence of grade ≥2 leukopenia, with a 14.4-fold increased risk compared to those treated in the prone position, even after multivariable adjustment. This disparity may be attributed to the reduced pelvic bone marrow irradiation volume in the prone position (23). Previous studies have demonstrated that active bone marrow (ABM) located in the iliac and sacral regions exhibits heightened sensitivity to low-dose radiation, with dosimetric parameters such as ABM-V10 and ABM-V20 being closely linked to hematologic toxicity (24–26). Although our study utilized conventional bone structure-based segmentation and identified no significant differences in dose-volume histograms (DVH) between positions, this method may underestimate the actual protective effect of prone positioning on functional marrow. A recent meta-analysis revealed that traditional pelvic DVH metrics, such as V10, can only account for a portion of the variability in hematologic toxicity (25). In contrast, imaging-defined ABM using 18F-FLT PET or IDEAL-IQ MRI, particularly ABM-V20, significantly enhances predictive accuracy (27, 28). Furthermore, we observed that lower baseline hemoglobin levels were associated with an increased risk of toxicity, while a higher pathological stage correlated with greater vulnerability, suggesting that both hematopoietic reserve and tumor burden should be considered in individualized positioning strategies.

In contrast to its hematologic advantage, the prone position was associated with less favorable outcomes concerning gastrointestinal toxicity. Although prone positioning increased low-dose exposure to bowel and rectum, the association with diarrhea approached but did not reach statistical significance in univariable analyses, and multivariable modeling suggested a protective effect for supine. Larger cohorts are required to precisely quantify this relationship. This finding is consistent with our dosimetric results, which indicated significantly higher relative volumes of the bowel bag and rectum exposed to low-dose radiation (V5–V15) in the prone group. Low-dose radiation may not directly induce structural injury, but it can indirectly precipitate symptoms such as diarrhea by compromising intestinal barrier function and eliciting inflammatory responses (29, 30). Moreover, individual factors such as postoperative adhesions and impaired gastrointestinal function may amplify the position-related effects of radiation on the bowel (31). These findings suggest that for patients undergoing concurrent chemoradiotherapy or those with a history of abdominal surgery or underlying gastrointestinal fragility, the supine position may offer a safer therapeutic profile.

This study possesses several methodological strengths. First, the use of propensity score matching and multivariable adjustment minimized baseline confounding factors and enhanced the robustness of the findings. Second, the integration of dosimetric analysis, toxicity profiles, and setup error data provides a comprehensive, multidimensional framework to inform position-related decision-making in postoperative radiotherapy.

Nonetheless, this study has several limitations. First, this retrospective, single-institution design may limit generalizability. Validation in prospective, multi-center cohorts with a broader case mix and diverse practice patterns will be essential to confirm and refine these risk-adaptive positioning recommendations. Second, hematologic risk was estimated from anatomic pelvic bone structures rather than functional marrow imaging (e.g., FLT-PET, MRI), which could more accurately delineate active marrow and strengthen toxicity prediction. Third, exclusive reliance on clinician grading may underestimate symptom burden; future prospective studies should incorporate validated PRO instruments. Fourth, our analyses focused on acute events; subacute and late gastrointestinal and hematologic effects were not captured and warrant standardized longitudinal evaluation. Finally, we did not evaluate adaptive planning across fractions (e.g., variable bladder filling and bowel motion), which may be especially relevant for prone positioning and warrants prospective investigation. Future research should integrate functional imaging, candidate biological or radiomic biomarkers, and adaptive radiotherapy platforms to refine individual susceptibility profiling, guide position-tailored planning and adaptation, and ultimately improve position-selection models toward more precise and safer radiotherapy strategies.

## Conclusions

This study demonstrates that the prone position effectively reduces hematologic toxicity and may be more suitable for patients with compromised baseline blood counts or a limited hematopoietic reserve. Conversely, the supine position appears to mitigate the risk of radiation-induced diarrhea and may be preferable for individuals with pre-existing gastrointestinal vulnerability or postoperative adhesions. Optimal positioning for postoperative radiotherapy in cervical cancer should extend beyond a dose-centric paradigm and adopt an individualized framework that incorporates the toxicity spectrum, baseline patient characteristics, and organ functional status.

## Data Availability

The raw data supporting the conclusions of this article will be made available by the authors, without undue reservation.

## References

[1] Bray F Laversanne M Sung H Ferlay J Siegel RL Soerjomataram I. Global cancer statistics 2022: GLOBOCAN estimates of incidence and mortality worldwide for 36 cancers in 185 countries. CA Cancer J Clin. (2024) 74:229–63. doi: 10.3322/caac.21834, PMID: 38572751

[2] N. National Comprehensive Cancer. NCCN Clinical Practice Guidelines in Oncology (NCCN Guidelines^®^): Cervical Cancer. Plymouth Meeting, PA: National Comprehensive Cancer Network (2025).

[3] Cibula D Raspollini MR Planchamp F Centeno C Chargari C Felix A. ESGO/ESTRO/ESP Guidelines for the management of patients with cervical cancer - Update 2023. Int J Gynecol Cancer. (2023) 33:649–66. doi: 10.1136/ijgc-2023-004429, PMID: 37127326 PMC10176411

[4] Lee SW Kim A Lee SJ Kim SH Lee JH. Intensity-modulated radiation therapy for uterine cervical cancer to reduce toxicity and enhance efficacy - an option or a must? A Narrat Rev Cancer Res Treat. (2024) 56:1–17. doi: 10.4143/crt.2023.562, PMID: 37654111 PMC10789959

[5] Anghel B Serboiu C Marinescu A Taciuc IA Bobirca F Stanescu AD. Recent advances and adaptive strategies in image guidance for cervical cancer radiotherapy. Med (Kaunas). (2023) 59:1735. doi: 10.3390/medicina5910, PMID: 37893453 PMC10608436

[6] Gonzalez VJ Hullett CR Burt L Rassiah-Szegedi P Sarkar V Tward JD. Impact of prone versus supine positioning on small bowel dose with pelvic intensity modulated radiation therapy. Adv Radiat Oncol. (2017) 2:235–43. doi: 10.1016/j.adro.2017.01.005, PMID: 28740937 PMC5514253

[7] Li C Xiao YP Huang L Jing W Zhang B Huang SH. High buttocks supine position to reduce small bowel exposure in gynecological radiotherapy. Radiat Oncol. (2024) 19:131. doi: 10.1186/s13014-024-02522-6, PMID: 39334494 PMC11428566

[8] Wang D Li B Chen L Li Z Kong F Yan H. Dosimetric effects of prone immobilization devices on skin in intensity-modulated radiation therapy for gynecologic cancer: a retrospective study. BMC Cancer. (2024) 24:1464. doi: 10.1186/s12885-024-13111-x, PMID: 39609758 PMC11603859

[9] Yan H Wu M Wang W Wang D Huang X Dong J. Dosimetry and acute radiation enteritis comparison between prone and supine position in IMRT for gynecological cancers. J Appl Clin Med Phys. (2023) 24:e14135. doi: 10.1002/acm2.14135, PMID: 37621141 PMC10691632

[10] Zheng Z Liu D Su Y. Supine/prone position fixation treatment in cervical cancer radiotherapy. J Cancer Res Ther. (2025) 21:401–8. doi: 10.4103/jcrt.jcrt_2050_24, PMID: 40317145

[11] Ge BB Liu Y Jin JH Wu JT Liu HT He CY. Effect of bladder filling status on positioning errors in post-hysterectomy cervical cancer radiotherapy. Ann Med. (2023) 55:2249936. doi: 10.1080/07853890.2023.2249936, PMID: 37683195 PMC10494734

[12] Banerjee R Chakraborty S Nygren I Sinha R. Small bowel dose parameters predicting grade ≥ 3 acute toxicity in rectal cancer patients treated with neoadjuvant chemoradiation: an independent validation study comparing peritoneal space versus small bowel loop contouring techniques. Int J Radiat Oncol Biol Phys. (2013) 85:1225–31. doi: 10.1016/j.ijrobp.2012.09.036, PMID: 23182394

[13] Stromberger C Kom Y Kawgan-Kagan M Mensing T Jahn U Schneider A. Intensity-modulated radiotherapy in patients with cervical cancer. An intra-individual comparison of prone and supine positioning. Radiat Oncol. (2010) 5:63. doi: 10.1186/1748-717X-5-63, PMID: 20598136 PMC2904783

[14] Pinkawa M Gagel B Demirel C Schmachtenberg A Asadpour B Eble MJ. Dose-volume histogram evaluation of prone and supine patient position in external beam radiotherapy for cervical and endometrial cancer. Radiother Oncol. (2003) 69:99–105. doi: 10.1016/S0167-8140(03)00244-5, PMID: 14597362

[15] Drzymala M Hawkins MA Henrys AJ Bedford J Norman A Tait DM. The effect of treatment position, prone or supine, on dose-volume histograms for pelvic radiotherapy in patients with rectal cancer. Br J Radiol. (2009) 82:321–7. doi: 10.1259/bjr/57848689, PMID: 19188240

[16] Wallrapp A Chiu IM. Neuroimmune interactions in the intestine. Annu Rev Immunol. (2024) 42:489–519. doi: 10.1146/annurev-immunol-101921-042929, PMID: 38941607 PMC13058849

[17] Jadon R Pembroke CA Hanna CL Palaniappan N Evans M Cleves AE. A systematic review of organ motion and image-guided strategies in external beam radiotherapy for cervical cancer. Clin Oncol (R Coll Radiol). (2014) 26:185–96. doi: 10.1016/j.clon.2013.11.031, PMID: 24566332

[18] Schmidhalter D Malthaner M Born EJ Pica A Schmuecking M Aebersold DM. Assessment of patient setup errors in IGRT in combination with a six degrees of freedom couch. Z fur Med Physik. (2014) 24:112–22. doi: 10.1016/j.zemedi.2013.11.002, PMID: 24418323

[19] Xu H Zhang Z Tian B Li X Bian Y Liang X. Evaluation of corrective effect of 6 degree of freedom couch on setup errors in intensity modulated radiotherapy for postoperative rectal cancer patients. Front Oncol. (2023) 13:1030599. doi: 10.3389/fonc.2023.1030599, PMID: 36816975 PMC9929531

[20] Bayley AJ Catton CN Haycocks T Kelly V Alasti H Bristow R. A randomized trial of supine vs. prone positioning in patients undergoing escalated dose conformal radiotherapy for prostate cancer. Radiother Oncol. (2004) 70:37–44. doi: 10.1016/j.radonc.2003.08.007, PMID: 15036850

[21] Mohamed R Elawadi AA Alkhanein N Alharth M Asiri M. Factors affecting isocenter displacement and planning target volume margin for patients with rectal cancer receiving radiation therapy. Adv Radiat Oncol. (2022) 7:101060. doi: 10.1016/j.adro.2022.101060, PMID: 36420207 PMC9677216

[22] Li J Kong X Cheng C Wang G Zhuang H Yang R. Comparison between supine and prone patient setup for lumbosacral spinal stereotactic body radiosurgery with CyberKnife. Front Oncol. (2023) 13:959447. doi: 10.3389/fonc.2023.959447, PMID: 37077832 PMC10106579

[23] Chopra S Gupta S Kannan S Dora T Engineer R Mangaj A. Late toxicity after adjuvant conventional radiation versus image-guided intensity-modulated radiotherapy for cervical cancer (PARCER): A randomized controlled trial. J Clin Oncol. (2021) 39:3682–92. doi: 10.1200/JCO.20.02530, PMID: 34506246

[24] Autorino R Cusumano D Rinaldi RM Giannini R De Luca V Campitelli M. Correlation between radiation dose to bone marrow subregions and acute hematologic toxicity inendometrial cancer treated with external beam radiotherapy. Clin Transl Radiat Oncol. (2025) 52:100942. doi: 10.1016/j.ctro.2025.100942, PMID: 40124647 PMC11927717

[25] Corbeau A Kuipers SC de Boer SM Horeweg N Hoogeman MS Godart J. Correlations between bone marrow radiation dose and hematologic toxicity in locally advanced cervical cancer patients receiving chemoradiation with cisplatin: a systematic review. Radiother Oncol. (2021) 164:128–37. doi: 10.1016/j.radonc.2021.09.009, PMID: 34560187

[26] Rahimy E von Eyben R Lewis J Hristov D Kidd E. Evaluating dosimetric parameters predictive of hematologic toxicity in cervical cancer patients undergoing definitive pelvic chemoradiotherapy. Strahlenther Onkol. (2022) 198:773–82. doi: 10.1007/s00066-021-01885-z, PMID: 35059758

[27] McGuire SM Bhatia SK Sun W Jacobson GM Menda Y Ponto LL. Using [(18)F]Fluorothymidine imaged with positron emission tomography to quantify and reduce hematologic toxicity due to chemoradiation therapy for pelvic cancer patients. Int J Radiat Oncol Biol Phys. (2016) 96:228–39. doi: 10.1016/j.ijrobp.2016.04.009, PMID: 27319286 PMC4982822

[28] Gao T Wei L Jiang L Ma S Zhang W Zhang Y. Dose-volume parameters of spared magnetic resonance imaging-defined active bone marrow predict hematologic toxicity in pelvic Malignancies patients undergoing radiotherapy: A cohort study. Technol Cancer Res Treat. (2024) 23:15330338241255283. doi: 10.1177/15330338241255283, PMID: 38752234 PMC11102680

[29] Lu L Li F Gao Y Kang S Li J Guo J. Microbiome in radiotherapy: an emerging approach to enhance treatment efficacy and reduce tissue injury. Mol Med Cambridge Mass. (2024) 30:105. doi: 10.1186/s10020-024-00873-0, PMID: 39030525 PMC11264922

[30] Park HY Yu JH. X-ray radiation-induced intestinal barrier dysfunction in human epithelial Caco-2 cell monolayers. Ecotoxicol Environ Saf. (2023) 264:115404. doi: 10.1016/j.ecoenv.2023.115404, PMID: 37625335

[31] Shadad AK Sullivan FJ Martin JD Egan LJ. Gastrointestinal radiation injury: symptoms, risk factors and mechanisms. World J Gastroenterol. (2013) 19:185–98. doi: 10.3748/wjg.v19.i2.185, PMID: 23345941 PMC3547560

