# Supplementary figures and images for: Comparative impact of supine vs prone positioning on dose distribution, acute toxicity, and setup error in postoperative radiotherapy for cervical cancer: a multidimensional propensity-matched cohort study
Source: Front Oncol. 2025 Oct 22;15:1637443. doi: 10.3389/fonc.2025.1637443 (PMC12585954; doi:10.3389/fonc.2025.1637443)

Supine position

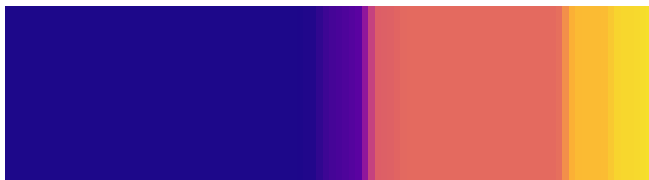

Dose (Gy)

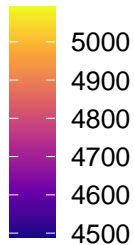

Prone position

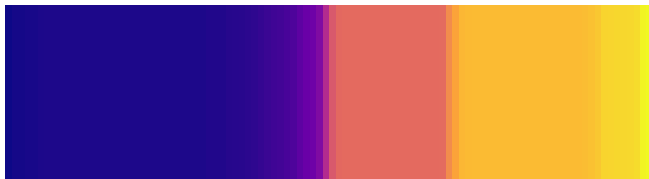

20% 40% 60% 80% 100%

Patients (percentile order)

Supplement: Supplementary file 1 [file DataSheet1.pdf]
